# The National Coordinated Citrien eHealth Program to Scale Up Telemonitoring: Protocol for a Before-and-After Evaluation Study

**DOI:** 10.2196/45201

**Published:** 2023-07-26

**Authors:** Harm Gijsbers, Azam Nurmohamed, Tom H van de Belt, Marlies Schijven, The Citrien 2 Project Leaders and Steering Group

**Affiliations:** 1 Department of Surgery Amsterdam UMC, location University of Amsterdam Amsterdam Netherlands; 2 Amsterdam Public Health Digital Health Amsterdam UMC Amsterdam Netherlands; 3 Amsterdam Gastroenterology and Metabolism Amsterdam UMC Amsterdam Netherlands; 4 Department of Internal Medicine Amsterdam UMC, location University of Amsterdam Amsterdam Netherlands; 5 Center for Sustainable Healthcare HAN University of Applied Sciences Nijmegen Netherlands; 6 see Acknowledgments

**Keywords:** study protocol, implementation, upscaling, telemonitoring, telemedicine, e-health, eHealth, healthcare, health care

## Abstract

**Background:**

Sustainable implementation of telemonitoring in health care is challenging, especially if one aims to scale up telemonitoring initiatives nationwide. The National collaborative eHealth program in the Netherlands is supporting the nationwide upscaling of telemonitoring in 3 clinical domains by implementing telemonitoring in all Dutch university medical centers (UMCs). The chosen telemonitoring concepts are (1) telemonitoring solutions in the domain of cardiology, (2) telemonitoring solutions providing care from a distance in obstetrics, and (3) telemonitoring solutions monitoring vital functions in hospital wards.

**Objective:**

The aim of this study is to evaluate the upscaling of telemonitoring in Dutch university hospitals in order to gain a better knowledge of the process, methods, and outcomes of nationwide upscaling strategies. Our hypothesis is that by the completion of the Citrien program’s scale-up, telemonitoring will be operational in all UMCs but not normalized in routine care.

**Methods:**

A before-and-after study will be conducted to assess upscaling. The theoretical frameworks used are the framework for nonadoption, abandonment, scale-up, spread, and sustainability; the Normalization Process Theory; and a project management tool Project Canvas. The primary outcome of the study is the degree of normalization to which health care providers at UMCs consider telemonitoring a part of their routine practice, measured using the Normalization MeAsurement Development tool (NoMAD). Our secondary outcome is the uptake of telemonitoring at the Dutch UMCs, using management data from UMCs’ business intelligence systems query.

**Results:**

Data will be collected between May 2020 and December 2022. Results were retrieved in June 2023. UMCs’ business intelligence systems are queried for data for the secondary outcome measures. There is a risk that the UMCs will not be able to provide this management information. The laws and regulations governing telemonitoring in the Netherlands are changing, with the Electronic Data Exchange in Health Care Act (Wet elektronische gegevensuitwisseling in de zorg) and the European Health Data Space Act expected to positively influence implementation and upscaling.

**Conclusions:**

The Citrien program is a nationally coordinated change management program that is scaling up telemonitoring across contexts and settings. This study will produce original data on the uptake and upscaling of telemonitoring at Dutch UMCs. Future initiatives to implement eHealth in the health care sector may be guided by the wide range of success factors, obstacles, and experiences collected through this program. The network itself may be of great value impacting future acceleration of eHealth initiatives.

**International Registered Report Identifier (IRRID):**

DERR1-10.2196/45201

## Introduction

Telemonitoring in health care can be defined as the use of IT to monitor a patient’s status remotely [1-3]. Telemonitoring includes the collection, transmission, evaluation, and communication of individual health data from a patient to their health care provider from outside a hospital to the hospital record using technology [3]. Telemonitoring may not only benefit health outcomes [4-6] but also reduce workload for clinical staff and save health care resources. [7]

Significant advantages for patients, the economy, and society could result from widespread telehealth adoption [8]. For example, using a digital platform for blood pressure and symptom monitoring in antenatal care for high-risk women is related to lower costs and equal health outcomes in conventional care [9].

Hence, sustainable adoption, implementation, and subsequent upscaling of telemonitoring initiatives in hospital settings remains challenging nationwide [10-12]. Although there are systematic reviews on adopting digital care [13,14], to our knowledge, little has been published on national implementation programs or national scale-up. A scoping review on nationwide upscaling of telemonitoring revealed heterogeneity in describing nationwide uptake. According to this study, there are 89 factors influencing nationwide upscaling [10].

Implementation of telemonitoring in the Dutch medical landscape is still rather limited, focusing on use in pilots and research settings [15,16]. To date, only 19% of the Dutch medical specialists report the use of telemonitoring in their daily practice [17]. Ten percent of the Dutch medical specialists claim that the COVID-19 pandemic has increased their use of telemonitoring [18]. Only 6% of Dutch patients have reported sharing their health data with their own hospital-based physician, and only 3% of them reported sharing their health data with their hospital-based nurse for the purpose of telemonitoring [17,19].

In the Netherlands, the Ministry of Health, Welfare and Sports (HWS) has called upon the university medical centers (UMCs) to take the lead in shaping the digital health landscape, ensuring sustainable health care [20]. Over the last decade, the Ministry of HWS continues to advocate this role for UMCs [21-23], and recent policy papers continue to do so [24]. From 2014, Dutch UMCs collaborated within the “Citrien” eHealth program, which was founded by ZonMw—a health research and development organization in the Netherlands [25]. The Citrien program is now in its second term. In the first term, the Citrien program was themed “Evidence-Based eHealth.” In this program, the effectiveness of eHealth was jointly studied [26]. This resulted in an increased focus on specific eHealth topics including monitoring and insights into the positive contribution of eHealth in health care and a nationwide eHealth vision [27-29]. Despite valuable projects and scientific evidence in the first term of the program (2014-2018), nationwide upscaling of these projects was not in scope. Therefore, a follow-up program was implemented, called “Citrien Implementation and Upscaling.” This is the current term of the Citrien program running between 2018 and 2022 [23,30]. At this time, the Citrien program aims at scaling up 3 telemonitoring concepts in a collaborative effort at all Dutch UMCs, including many of their partnering hospitals and systems.

The UMCs in the Netherlands—8 in 2019 and 7 at the moment—act as a knowledge and network partner for nonuniversity hospitals. An effective scaling up of telemonitoring (and obtained knowledge regarding scaling up) at UMCs could affect roughly 70 additional nonuniversity hospitals in the Netherlands. Within this program, a strong implementation network has emerged, aiding UMCs in being more successful in jointly scaling up telemonitoring with other hospitals and care settings [31].

The aim of this study is to evaluate the upscaling of telemonitoring in Dutch university hospitals in order to gain a better knowledge of the process, methods, and outcomes of nationwide upscaling strategies.

Our hypothesis is that by the completion of the Citrien program’s scale-up, telemonitoring will be operational at all UMCs, although it has yet to become normal practice among health care providers.

## Methods

### Study Design

To evaluate the success in scaling up of 3 telemonitoring concepts across settings, flexibility in vendor and concept selection is deemed necessary. This is required to better adapt to existing variations in hospital settings from a technical, organizational, financial, and cultural perspective while simultaneously safeguarding outcome end goals. A before-and-after study will be conducted to assess the degree of successful upscaling.

### Theoretical Frameworks

In order to describe essential elements of the Citrien program—including the intervention, target group, application design, and implementation choices—the framework for nonadoption, abandonment, scale-up, spread, and sustainability (NASSS) will be used [32]. The NASSS framework consists of 7 domains and is designed to identify activities such as those performed by Citrien within a complex context from local adoption to sustainable national scale-up. This framework is considered to be a good fit, as it intends to be used reflexively to guide conversations and help generate ideas about barriers and facilitators [33]. The NASSS framework is intended to evaluate unfolding technology programs in real time and, in particular, to identify and manage their emergent uncertainties and interdependencies. In our study, the NASSS framework guides the estimation of the complexity of implementation and subsequent scale-up.

Given that this protocol describes the scaling up of 3 telemonitoring projects at 8 university hospitals, the descriptions of all of its essential elements are very lengthy. We, therefore, have appropriately explained the analysis of key elements in accordance with the NASSS framework in Multimedia Appendices 1-3.

Whether end users (health care providers and patients) integrate telemonitoring into daily care (normalization) is a socio-technical issue. An implementation framework such as the NASSS does not provide sufficient insight. To measure normalization, we, therefore, have used the Normalization MeAsurement Development (NoMAD) questionnaire, which is based on Normalization Process Theory (NPT). According to the NPT, a normalization process is a result of individual and group behaviors and entails the embedding and integration of health care innovations into everyday treatment.

According to the theory, 4 constructs play a central role in generating the work of implementation:

- Coherence of the innovation with the goals of daily routine
- Cognitive participation as a process of enrollment and engagement of individual participants and groups
- Collective action by individuals and groups to apply the innovation in daily routine
- Reflexive monitoring through which participants in the implementation process evaluate and appraise the use of the innovation in practice

The NPT successfully aids in the conceptual understanding of implementation processes and outcomes across a wide variety of health care settings [34].

The brief self-report NoMAD questionnaire was designed in accordance with the general approach of the NPT for the goal of detecting factors likely to influence normalization processes [35]. The questionnaire aims to (1) facilitate the evaluation of normalization progress over time in an implementation project and (2) facilitate the comparison of normalization (progress or outcomes) among sites in multicenter studies. The NoMAD questionnaire has been translated to Dutch [36]. Therefore, the NoMAD questionnaire seems suitable as a tool for tracking the progress of implementation for telemonitoring in our Citrien program.

**Figure 1:**
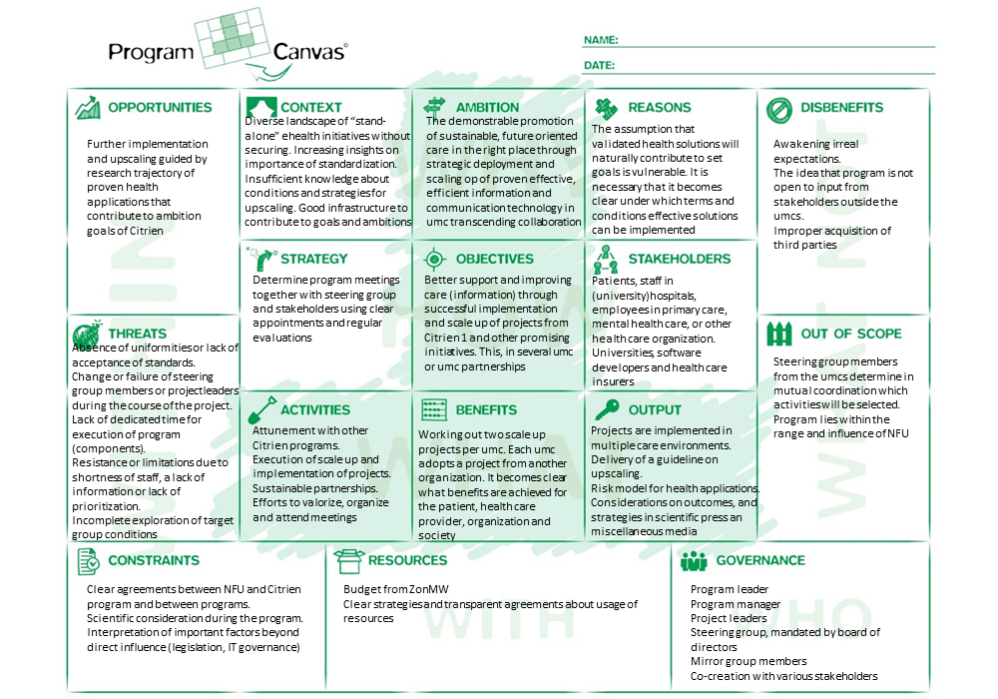
Citrien program canvas [37].

A project-based approach (Program Canvas; Figure 1), advocated by the Netherlands Federation of University Medical Centres, was chosen as the management template for the Citrien program. Program Canvas, and the associated Project Canvas, is a simple model consisting of 16 elements from which a program is built. Project Canvas provides a universal technology to design and initiate new projects. The Canvas has been particularly created for teams involved in interdisciplinary projects, who aim at developing a common ground in an early stage of the project [37].

### Settings and Intervention

In 2020, a total of 1,200,000 patients were treated in all of the UMCs in total [38]. The telemonitoring concepts aim to encourage and foster adoption of (1) telemonitoring solutions in the domain of cardiology, (2) telemonitoring solutions providing care from a distance in obstetrics, and (3) telemonitoring solutions monitoring vital functions in hospital wards. In a prior Citrien initiative, these telemonitoring concepts were developed and evaluated [9,29,39-43].

**Figure 2:**
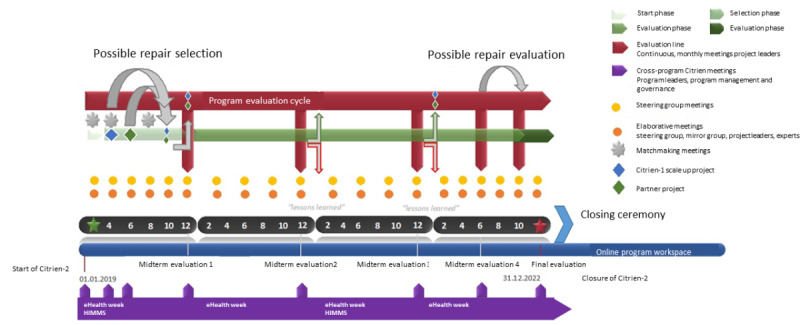
Program planning and characteristics. HIMMS: Healthcare Information and Management Systems Society.

The Citrien program was structured such that each UMC appointed a tripod of a steering group member, a deputy steering group member, and a project leader. Members are all professionals working at the UMCs, with expertise in the field of eHealth. The steering group members were formally appointed by their hospital boards, and the other members of the tripod were subsequently chosen by the steering group members. All members are in a key position or a management role to guide implementation within their organization. The Citrien program characteristics are outlined in Textbox 1 and Figure 2.

**Outline of the Citrien program.**

**The Citrien eHealth Program**

The Citrien eHealth program focuses on nationwide upscaling of telemonitoring by actual implementation at university medical centers (UMCs) in the Netherlands. The diverse concept of telemonitoring among cardiology patients, high-risk pregnancies, and for in-patients undergoing continuous monitoring of vital signs using wearables is implemented and researched. For all concepts chosen, differences in the target population or chosen technology (or both) may exist to fit the existing infrastructures and hospital strategy. In the Netherlands, different electronic health records (EHR) are in use by the UMCs. To facilitate interoperability, different requirements must be met to accommodate upscaling of the concepts. UMCs were free to opt for stand-alone solutions, although embedding technology into the existing EHR is a well-known enabler for implementation. Although the technology supply model is open to UMCs, the participating UMCs had to comply to the vision of the Citrien program, choosing trusted suppliers with validated, reliable, secure, and scalable solutions.

Collaboration within the Citrien program supported the project leaders in preparing a business case for their own organization. To analyze barriers and enablers for scaling up telemonitoring locally, UMCs used methodologies they were most experienced with. The Citrien program supported the analysis and drafting of implementation strategies by exchanging best practices among project leaders.

This Citrien program is aligned with relevant initiatives such as information standards and regulations on secure exchange of health data. Part of the program’s strategy is to strengthen and foster cooperation within the UMCs and to foster cross-UMC partnerships while implementing various telemonitoring initiatives. Overarching issues related to scaling up such as reimbursement, screening new technologies for viability and suitability, procurement of devices, and privacy aspects were tackled by the UMCs in a joint effort. Typical of the Citrien program was the monitoring of change over a period of 2 years. Project leaders kept track of progress every month during their meetings.

### Outcomes and Data Collection

#### Overview

Upscaling of telemonitoring is operationalized as the actual use (uptake) and normalization of telemonitoring. Effectiveness is expressed in the degree of uptake and normalization of the use of telemonitoring at the UMCs. Data will be aggregated in a protective collaborative web-based environment created for this purpose and managed by the chair of the steering group (MS) and the principal investigator (HG).

#### Primary Outcome

The primary outcome is the degree of normalization to which health care providers consider telemonitoring to be a part of their routine practice, as measured by the NoMAD questionnaire [36]. The NoMAD questionnaire has high internal consistency and has been validated in heterogeneous examples in different languages and settings [35,36].

#### Recruitment of Respondents

We make a pragmatic choice for a purposeful sample of respondents without performing a power calculation. Citrien program project leaders within each UMC recruited participants for each telemonitoring project to take part in the survey. The local project leader within each UMC sent a standardized invitation email to 10 self-selected health care professionals involved in the adoption of a telemonitoring project, containing the objective of the study and a link to a web-based survey. We aimed for a total of 10 respondents per project and consequently 30 respondents per UMC. This is a pragmatic choice because project leaders have noted at this stage of the scale-up that not more than 10 care providers are involved in the telemonitoring projects. Three reminders were sent. The local project leaders and the researcher were both unable to determine who had filled out the questionnaire. The questionnaire did not collect any email or IP addresses. The questionnaire responses could not be linked to specific individuals.

#### Secondary Outcome

The secondary outcome is the uptake of telemonitoring in Dutch UMCs, as measured by (1) the absolute number of patients using telemonitoring within each UMC and (2) the percentage of patients using telemonitoring compared to the number of patients eligible for telemonitoring.

These data are management data or business intelligence data. Patients are not actively being included. This study observes the nationwide coordination of implementation and scale-up practices carried out on a daily basis.

### Statistical Analysis

The results will be presented as a summary of the outcome measures sorted by UMC and telemonitoring project, using frequencies, medians, and percentages for categorical factors and means and SDs for continuous measures in order to have as much information as possible available for exploration and nonparametric analysis. For evaluation of NoMAD data, we will use the Wilcoxon signed-rank test. To evaluate the secondary outcome measures within each UMC, we will use a 2-tailed paired *t* test. All statistical analyses will be conducted using SPSS software (version 28.0, 2021 release; IBM Corp).

### Ethical Considerations

The primary outcome measure is a questionnaire administered to health care professionals. It is a nonburdensome questionnaire that is completed voluntarily and anonymously. Health care professionals are not subjected to any actions, nor are any behavioral rules imposed.

The secondary outcome measure is management information collected retrospectively. No data are collected from patients, and no actions or behavioral rules are imposed on patients. The use of telemonitoring is always and only in the context of patient care. The reason for monitoring is not different but only the method of delivery is in a digital manner.

The Medical Ethics Review Committee Amsterdam UMC exempted this study from ethical review. The study protocol is registered on the Open Science Framework [44].

## Results

The baseline assessment was carried out from May 2020 to January 2021. Data on scaling up are collected quarterly. The final results were retrieved in June 2023. Data collection will be carried out under governance of the project leaders at every UMC. The findings are likely to be available in the second half of 2023.

## Discussion

### Anticipated Findings

This study will define the degree of uptake of telemonitoring at Dutch UMCs and elucidate how it is normalized in the daily routine of health care professionals. This protocol is important as it describes the theoretical framework that will be used and activities that will be performed. Evaluating the implementation of 3 telemonitoring projects at all university hospitals of the Netherlands provides the opportunity to provide a better understanding of the process, methods, and outcomes of a nationwide implementation of strategies.

### Limitations

While launching this initiative, we thoroughly analyzed the scale-up’s challenges and created tailored implementation plans and strategies for each UMC. However, we are unable to completely control all variables and influences in this real-world evaluation study. This implies that there remains a degree of uncertainty about the effectiveness of the scale-up program. If unforeseen circumstances arise despite carefully planned implementation actions and plans, the effect of the Citrien program may be underestimated. Thus, maybe the impact of the Citrien program could be overestimated if there are favorable changes in the health care environment that are unrelated to the Citrien program and accelerate the scale-up of telemonitoring. Nonetheless, it is interesting to evaluate this in a systematic approach.

Although not likely, limitations may also occur as a result of data collection; for example, when too few responses are received for the primary outcome measure. We can send additional reminders and request project leaders to exercise leadership in this situation.

The UMCs’ business intelligence systems are queried for data for the secondary outcome measures. Although the outcome measures are actual fundamental parameters, there is a risk that the UMCs will not be able to provide this management information. It is crucial to acknowledge this and communicate the lesson that has been taught if this happens.

Nationwide upscaling of telemonitoring in different target groups requires a range of technologies and suppliers. The Citrien program aims to contribute to important preconditions, such as writing a business case, providing insight into barriers and enablers, and designing implementation strategies. With a pragmatic approach, Citrien aims to provide insight in, and solutions for, various barriers such as interoperability, reimbursements, adjustments in care paths, privacy policies, and procurement policies. These initiatives were carefully and purposefully developed in light of the variables that affect nationwide upscaling, which were identified in a recent scoping review on upscaling telemonitoring [10].

The laws and regulations governing the use of telemonitoring in the Netherlands are subject to change. The legal responsibility in monitoring patients remotely was still unclear at the start of this program. From 2023, the Electronic Data Exchange in Health Care Act (Wet elektronische gegevensuitwisseling in de zorg) mandates digital data exchange among health care providers. This also affects the processing of telemonitoring data. In the near future, European Health Data Space Act will also be enacted [45]. These regulations are expected to positively influence implementation and upscaling.

Finally, there are other eHealth scale-up initiatives in the Netherlands. In order to encourage eHealth activities outside of hospitals, the government has incentive regulations for eHealth in primary care [46]. A coalition of patients, health care professionals, health insurers, and suppliers was formed by the Netherlands Patient Federation in order to advance digital care [47]. Although these programs are not aimed at UMCs, the Citrien program will include similar strategies.

### Conclusions

The Citrien program is a nationally coordinated change management program in the eHealth domain, now scaling up telemonitoring across situations and settings. We expect that this study will yield unique evidence about the use and upscaling of telemonitoring in Dutch UMCs. This study protocol could serve as inspiration for others who would like to implement a similar programmatic intervention for nationwide scale-up of applications, such as a digital ward or artificial intelligence–driven decision support. The success factors, barriers, and experiences collected in this program could guide future projects that aim to implement eHealth in the health care sector. The Citrien network itself may be of great value impacting future acceleration of eHealth initiatives.

## Appendix

Multimedia Appendix 1 NASSS framework analysis of telemonitoring in cardiac diseases. NASSS: nonadoption, abandonment, scale-up, spread, and sustainability.

Multimedia Appendix 2 NASSS framework analysis of telemonitoring in antenatal care. NASSS: nonadoption, abandonment, scale-up, spread, and sustainability.

Multimedia Appendix 3 NASSS framework analysis of telemonitoring vital signs. NASSS: nonadoption, abandonment, scale-up, spread, and sustainability.

## Abbreviations

HWS: Health, Welfare and Sports
NASSS: nonadoption, abandonment, scale-up, spread, and sustainability
NoMAD: Normalization MeAsurement Development
NPT: Normalization Process Theory
UMC: university medical center

## Data Availability

The data set that will be generated during this study are available from the corresponding author upon reasonable request.

## References

[1] Meystre S (2005). The current state of telemonitoring: a comment on the literature. Telemed J E Health.

[2] Field MJ, Institute of Medicine (1996). Telemedicine: A Guide to Assessing Telecommunications for Health Care.

[3] (2020). Telehealth: Defining 21st Century Care. American Telemedicine Association.

[4] Khalil A, Perry H, Lanssens D, Gyselaers W (2019). Telemonitoring for hypertensive disease in pregnancy. Expert Rev Med Devices.

[5] Jang S, Kim Y, Cho W (2021). A systematic review and meta-analysis of telemonitoring interventions on severe COPD exacerbations. Int J Environ Res Public Health.

[6] Kitsiou S, Paré Guy, Jaana M (2015). Effects of home telemonitoring interventions on patients with chronic heart failure: an overview of systematic reviews. J Med Internet Res.

[7] León Maria Alejandra, Pannunzio V, Kleinsmann M (2022). The impact of perioperative remote patient monitoring on clinical staff workflows: scoping review. JMIR Hum Factors.

[8] Hafner M, Yerushalmi E, Dufrense E, Gkousis E (2021). The potential socio-economic impact of telemedicine in Canada. RAND Corporation.

[9] van den Heuvel JF, van Lieshout C, Franx A, Frederix G, Bekker MN (2021). SAFE@HOME: Cost analysis of a new care pathway including a digital health platform for women at increased risk of preeclampsia. Pregnancy Hypertens.

[10] Gijsbers H, Feenstra TM, Eminovic N, van Dam D, Nurmohamed SA, van de Belt T, Schijven MP (2022). Enablers and barriers in upscaling telemonitoring across geographic boundaries: a scoping review. BMJ Open.

[11] Faber S, van Geenhuizen M, de Reuver M (2017). eHealth adoption factors in medical hospitals: a focus on the Netherlands. Int J Med Inform.

[12] Kristensen MBD, Høiberg Lone, Nøhr Christian (2019). Updated mapping of telemedicine projects in Denmark. Stud Health Technol Inform.

[13] Varsi C, Solberg Nes L, Kristjansdottir OB, Kelders SM, Stenberg U, Zangi HA, Børøsund Elin, Weiss KE, Stubhaug A, Asbjørnsen Rikke Aune, Westeng M, Ødegaard Marte, Eide H (2019). Implementation strategies to enhance the implementation of eHealth programs for patients with chronic illnesses: realist systematic review. J Med Internet Res.

[14] Simblett S, Greer B, Matcham F, Curtis H, Polhemus A, Ferrão José, Gamble P, Wykes T (2018). Barriers to and facilitators of engagement with remote measurement technology for managing health: systematic review and content analysis of findings. J Med Internet Res.

[15] Wouters M, Huygens M, Voogdt H, Meurs M, Groot J, de Bruin K, de Brabers A, Hofstede C, Friele R, van Gennip L (2019). Samen aan zet! eHealth-monitor 2019. Nivel.

[16] Wouters M (2019). Together we can do it! eHealth-monitor 2019. Self-management and telemonitoring theme discussion.

[17] (2022). E-healthmonitor 2021 Stand van zaken digitale zorg. Rijksinstituut voor Volksgezondheid en Milieu.

[18] van der Vaart R, Kouwenberg LHJA, Oosterhoff M, Rotteveel AH, van Tuyl L, van Vliet ED (2022). Ontwikkelingen rondom e-health tijdens de COVID-19-pandemie. Bevindingen vanuit de literatuur en empirisch onderzoek. Rijksinstituut voor Volksgezondheid en Milieu.

[19] (2022). Tabellenbijlage Nationaal Panel Chronisch zieken en Gehandicapten. Rijksinstituut voor Volksgezondheid en Milieu.

[20] Bruins, B.J. (2019). 33 278 Interdepartementaal Beleidsonderzoek: Universitair Medische Centra (UMC's) Nr. 8. Overheid.nl.

[21] Schippers, E.I. (2014). Kamerbrief ‘e-health en zorgverbetering’. https://www.tweedekamer.nl/kamerstukken/brieven_regering/detail?did=2014D25448&id=2014Z12697.

[22] (2017). Kamerbrief over digitale vaardigheden en zorginnovatie. Rijksoverheid.

[23] Bruins B (2019). Eindevaluatie Citrienfonds 2014-2018. Tweede Kamer der Staten-Generaal.

[24] (2022). Monitor umc’s 2021: Basiszorg in umc’s en inzet op de maatschappelijke opgaven. Nederlandse Zorgautoriteit.

[25] A good health for everybody. ZonMw.

[26] Citrien-1: Evidence Based E-health. Nederlandse Federatie van Universitair Medische Centra.

[27] Rauwerdink A, Kasteleyn MJ, Haafkens JA, Chavannes NH, Schijven MP, steering committee, of the Citrien fund program eHealth (2020). A national eHealth vision developed by University Medical Centres: a concept mapping study. Int J Med Inform.

[28] e-Health. Nederlandse Federatie van Universitair Medische Centra.

[29] Rauwerdink A, Kasteleyn MJ, Chavannes NH, Schijven MP (2021). Successes of and lessons from the First Joint eHealth Program of the Dutch University Hospitals: evaluation study. J Med Internet Res.

[30] Citrien-2: Implementatie en opschaling. Nederlandse Federatie van Universitair Medische Centra.

[31] Wouters M, Swinkels I, van Lettow B, de Jong J, Sinnige J, Brabers A, Friele R, van Gennip L (2018). E-health in verschillende snelheden: eHealth-monitor 2018. Nivel.

[32] Greenhalgh T, Abimbola S (2019). The NASSS Framework - a synthesis of multiple theories of technology implementation. Stud Health Technol Inform.

[33] Greenhalgh T, Wherton J, Papoutsi C, Lynch J, Hughes G, A'Court C, Hinder S, Fahy N, Procter R, Shaw S (2017). Beyond adoption: a new framework for theorizing and evaluating nonadoption, abandonment, and challenges to the scale-up, spread, and sustainability of health and care technologies. J Med Internet Res.

[34] May CR, Cummings A, Girling M, Bracher M, Mair FS, May CM, Murray E, Myall M, Rapley T, Finch T (2018). Using Normalization Process Theory in feasibility studies and process evaluations of complex healthcare interventions: a systematic review. Implement Sci.

[35] Rapley T, Girling M, Mair FS, Murray E, Treweek S, McColl E, Steen IN, May CR, Finch TL (2018). Improving the normalization of complex interventions: part 1 - development of the NoMAD instrument for assessing implementation work based on normalization process theory (NPT). BMC Med Res Methodol.

[36] Vis C, Ruwaard J, Finch T, Rapley T, de Beurs D, van Stel H, van Lettow B, Mol M, Kleiboer A, Riper H, Smit J (2019). Toward an objective assessment of implementation processes for innovations in health care: psychometric evaluation of the Normalization Measure Development (NoMAD) Questionnaire among mental health care professionals. J Med Internet Res.

[37] van der Tak T, Prevaas B, Cremer H (2016). Program Canvas. Samen naar de kern van je programma. Good Work Company.

[38] (2019). Facts and figures. Nederlandse Federatie van Universitair Medische Centra.

[39] Kauw D, Koole MA, Winter MM, Dohmen DA, Tulevski II, Blok S, Somsen GA, Schijven MP, Vriend JW, Robbers-Visser D, Mulder BJ, Bouma BJ, Schuuring MJ (2019). Advantages of mobile health in the management of adult patients with congenital heart disease. Int J Med Inform.

[40] Koole MAC, Kauw D, Winter MM, Dohmen DAJ, Tulevski II, de Haan R, Somsen GA, Schijven MP, Robbers-Visser D, Mulder BJM, Bouma BJ, Schuuring MJ (2019). First real-world experience with mobile health telemonitoring in adult patients with congenital heart disease. Neth Heart J.

[41] van den Heuvel JF, Lely AT, Huisman JJ, Trappenburg JC, Franx A, Bekker MN (2020). SAFE@HOME: Digital health platform facilitating a new care path for women at increased risk of preeclampsia - A case-control study. Pregnancy Hypertens.

[42] Breteler MJM, Huizinga E, van Loon K, Leenen LPH, Dohmen DAJ, Kalkman CJ, Blokhuis TJ (2018). Reliability of wireless monitoring using a wearable patch sensor in high-risk surgical patients at a step-down unit in the Netherlands: a clinical validation study. BMJ Open.

[43] Leenen JPL, Leerentveld C, van Dijk JD, van Westreenen HL, Schoonhoven L, Patijn GA (2020). Current evidence for continuous vital signs monitoring by wearable wireless devices in hospitalized adults: systematic review. J Med Internet Res.

[44] Gijsbers H (2022). Evaluation of the Citrien eHealth program for Nationwide upscaling of telemonitoring: A study protocol. Open Science Framework.

[45] European Health Data Space. European Commission.

[46] Stimuleringsregeling E-Health Thuis (SET). Rijksdienst voor Ondernemend Nederland.

[47] Vliegwiel, aanjagers van digitale transformatie in de zorg. Vliegwiel.

