# Supplementary material for: The National Coordinated Citrien eHealth Program to Scale Up Telemonitoring: Protocol for a Before-and-After Evaluation Study
Source: JMIR Res Protoc. 2023 Jul 26;12:e45201. doi: 10.2196/45201 (PMC10413225; doi:10.2196/45201)
Supplement: Multimedia Appendix 1 [file resprot_v12i1e45201_app1.docx]

**Appendix 1. NASSS framework analysis of telemonitoring in cardiac diseases. (by example of Greenhalgh et al 2017, table 2 )**

|  | Case A | Case B | Case C | Case D | Case E | Case F | Case G | Case H |
| --- | --- | --- | --- | --- | --- | --- | --- | --- |
| Domain 1. The condition or illness | | | | | | | | |
| 1A. Wat is the nature of the condition or illness? | Simple: Pts with grown up congenital heart disease and cardiac arrhythmias | Simple: Pts with myocard infarcts, atrial fibrillation, congenital heart diseases or after cardiac surgery | Complicated: Pts with heart failure | Complicated: Pts with cardiac arrhythmias | Complex: Pts with heart failure | Pts with heart failure | Pts with heart failure and pulmonary hypertension | Pts with cardiac arrhythmias |
| 1B. What are the relevant socio-cultural factors and comorbidities? | Simple: Relatively young, good eHealth literacy | Complicated: Mixed eHealth literacy | Complicated: Mixed eHealth literacy | Simple: Relatively young, good eHealth literacy | Complicated: Mixed eHealth literacy | Complicated: Mixed eHealth literacy | Simple: Mixed eHealth literacy | Complicated: Relatively young, less eHealth literacy |
| Domain 2 The Technology | | | | | | | | |
| 2A. What are the key features of the technology? | Complex: A biosensor with bluetooth linked to a smartphone. Not yet embedded in complex technical system | Simple: Several devices for rhythm, blood pressure, weight. With Bluetooth linked to a smartphone. Already in operation. | Simple: Mhealth using PPG on a smartphone. Stand alone system. | Simple: A 1leads ECG device linked to a smartphone, linked to a dataplatform. Already in operation. | Complicated: Accelerometry device linked to a smartphone. Not fully interoperable. | Complex: A 1leads ECG device linked to a smartphone, linked to a dataplatform. Not yet in operation. | Simple: A biosensor wirelessely linked to a data platform and app. Already in operation | Simple: A 1leads ECG device linked to a smartphone, linked to a dataplatform. Already in operation. |
| 2B. What kind of knowledge does the technology bring into play? | Simple: Same knowledge for reading ECG. Different care planning and control. | Simple: Same knowledge for reading ECG or BP. Different care planning and control. | Simple: PPG technology is different from ECG, but with similar outcome (cardiac rhythm). | Simple: Same knowledge for reading ECG. Different care planning and control. | Simple: Objective measurement of physical activity. Patients need education in order to selfmanage their lifestyle. | Simple: Same knowledge for reading ECG. Different care planning and control. | Simple: Technology allows for early intervention based on trends in BP | Simple: Same knowledge for reading ECG. Different care planning and control. |
| 2C. What knowledge and/or support is required to use the technology? | Complicated: Medical and technical support by a medical service centre. 24/7 support possible. | Complicated: Different care planning and control in the outpatient clinic. | Complicated: More frequent monitoring by cardiologists or nurse, based on incoming data. | Complicated: Medical and technical support by a medical service centre. Weekdays 9-5 support possible. | Complicated: Different care planning and control in the outpatient clinic. | Complicated: Medical and technical support by a medical service centre. Weekdays 9-5 support possible. | Complicated: More frequent monitoring by cardiologists, based on incoming data. | Complicated: Medical and technical support by a medical service centre. Weekdays 9-5 support possible. |
| 2D. What is the technology supply model? | Complicated: Third party and front-end integration in EHR. | Complicated: Third party and back-end integration in EHR. | Complicated: Third party and front-end integration in EHR. | Complicated: Third party and front-end integration in EHR. | Complicated: Third party and front-end integration in EHR. | Complicated: Third party and front-end integration in EHR. | Complicated: Third party and front-end integration in EHR. | Complicated: Third party and front-end integration in EHR. |
| Domain 3 The value proposition | | | | | | | | |
| 3A. What is the developer’s business case for the technology (supply-side value)? | unknown | unknown | unknown | unknown | unknown | unknown | unknown | unknown |
| 3B. What is its desirability, efficacy, safety and cost-effectiveness (demand-side value)? | Simple: fewer emergency admissions, fewer hospital admissions, fewer regular check-up appointments, more self-management for the patient | Simple: fewer emergency admissions, fewer hospital admissions, fewer regular check-up appointments, more self-management for the patient | Simple: fewer hospital admissions, fewer regular check-up appointments, more self-management for the patient | Simple: fewer emergency admissions, fewer hospital admissions, fewer regular check-up appointments, more self-management for the patient | Simple: Patient self-management and more active lifestyle. | Simple: fewer emergency admissions, fewer hospital admissions, fewer regular check-up appointments, more self-management for the patient | Simple: fewer hospital admissions, fewer regular check-up appointments, more self-management for the patient | Simple: fewer emergency admissions, fewer hospital admissions, fewer regular check-up appointments, more self-management for the patient |
| Domain 4 The adopter system (Staff, patient, lay carers) | | | | | | | | |
| 4A. What changes in staff roles, practices and identities are implied? | Complicated: less face-to-face contact, patient self-administered measurement, monitoring centralised, shift in tasks and skills of nurses | Complicated: less face-to-face contact, patient self-administered measurement, monitoring centralised, shift in tasks and skills of nurses | Complicated: less face-to-face contact, patient self-administered measurement, monitoring centralised, shift in tasks and skills of nurses | Complicated: less face-to-face contact, patient self-administered measurement, monitoring outsourced, shift in tasks and skills of nurses | Complicated: less face-to-face contact, patient self-administered measurement, monitoring centralised, shift in tasks and skills of nurses | Complicated: less face-to-face contact, patient self-administered measurement, monitoring centralised, shift in tasks and skills of nurses | Complicated: less face-to-face contact, implanted measurement, monitoring centralised, shift in tasks and skills of nurses | Complicated: less face-to-face contact, patient self-administered measurement, monitoring outsourced, shift in tasks and skills of nurses |
| 4B. What input is expected of the patient (and/or immediate carer) – and is this achievable by, and acceptable to, them? | Complicated: Technical skills for device pairing, patient self-administered measurement, responsibility or self-management among patients = achievable and acceptable | Complicated: Technical skills for device pairing, patient self-administered measurement, responsibility or self-management among patients = achievable and acceptable | Complicated: Technical skills for device pairing, patient self-administered measurement, responsibility or self-management among patients = achievable and acceptable | Complicated: Technical skills for device pairing, patient self-administered measurement, responsibility or self-management among patients = achievable and acceptable | Complicated: Technical skills for device pairing, patient self-administered measurement, responsibility or self-management among patients = achievable and acceptable | Complicated: Technical skills for device pairing, patient self-administered measurement, responsibility or self-management among patients = achievable and acceptable | Simple: No input expected = achievable and acceptable | Complicated: Technical skills for device pairing, patient self-administered measurement, responsibility or self-management among patients = achievable and acceptable |
| 4C. What is assumed about the extended network of lay carers? | Simple: Nothing in particular | Simple: Nothing in particular | Simple: Nothing in particular | Simple: Nothing in particular | Simple: Nothing in particular | Simple: Nothing in particular | Simple: Nothing in particular | Simple: Nothing in particular |
| Domain 5 The organization | | | | | | | | |
| 5A. What is the organization’s capacity to innovate? | Simple: Positive attitude management and healthcare providers. Sufficient (research) funding available for technology introduction Local champions in place. | Simple: Positive attitude management and healthcare providers. Sufficient (research) funding available for technology introduction Local champions in place. | Simple: Positive attitude management and healthcare providers. Sufficient (research) funding available for technology introduction Local champions in place. | Complicated: Positive attitude management and healthcare providers. Local champions in place. But limited resources. | Simple: Positive attitude healthcare providers. Sufficient (research) funding available for technology introduction Local champions in place. | Simple: Positive attitude healthcare providers. Sufficient (research) funding available for technology introduction Local champions in place. | Simple: Positive attitude management and healthcare providers. Sufficient (research) funding available for technology introduction Local champions in place. | Complicated: Positive attitude management and healthcare providers. Local champions in place. But limited resources. |
| 5B. How ready is the organization for this technology-supported change? | Simple: Organisation-wide innovation programme available at inception. A readiness analysis has been conducted but not openly available. | Simple: Organisation-wide innovation programme available at inception. | Simple: Organisation-wide innovation programme available at inception. A readiness analysis has been conducted but not openly available. | Complicated: Organisation-wide innovation programme not available at inception. A readiness analysis has not been conducted. | Simple: Organisation-wide innovation programme available at inception. A readiness analysis has been conducted but not openly available. | Complicated: Organisation-wide innovation programme not available at inception. A readiness analysis has not been conducted. | Simple: Organisation-wide innovation programme available at inception. A readiness analysis has been conducted but not openly available. | Complicated: Organisation-wide innovation programme not available at inception. A readiness analysis has not been conducted. |
| 5C. How easy will the adoption and funding decision be? | Complicated: no structural reimbursement, cost-benefit balance neutral or unknown. | Complicated: no structural reimbursement, cost-benefit balance neutral or unknown. | Complicated: no structural reimbursement, cost-benefit balance neutral or unknown. | Complicated: no structural reimbursement, cost-benefit balance neutral or unknown. | Complicated: no structural reimbursement, cost-benefit balance neutral or unknown. | Complicated: no structural reimbursement, cost-benefit balance neutral or unknown. | Complicated: no structural reimbursement, cost-benefit balance neutral or unknown. | Complicated: no structural reimbursement, cost-benefit balance neutral or unknown. |
| 5D. What changes will be needed in team interactions and routines? | Complicated: New routines and care pathways, new communication and escalation protocols, but readily aligned. | Complicated: New routines and care pathways, new communication and escalation protocols, but readily aligned. | Complicated: New routines and care pathways, new communication and escalation protocols, but readily aligned. | Complicated: New routines and care pathways, new communication and escalation protocols, but readily aligned. | Complicated: New routines and care pathways, new communication and escalation protocols, but readily aligned. | Complicated: New routines and care pathways, new communication and escalation protocols, but readily aligned. | Simple: no new team routines necessary. | Complicated: New routines and care pathways, new communication and escalation protocols, but readily aligned. |
| 5E. What work is involved in implementation and who will do it? | Complicated: Implementation analysis, defining implementation strategies, implementation / project planning. All activities coordinated by project leaders of the Citrien program. | Complicated: Implementation analysis, defining implementation strategies, implementation / project planning. All activities coordinated by project leaders of the Citrien program. | Complicated: Implementation analysis, defining implementation strategies, implementation / project planning. All activities coordinated by project leaders of the Citrien program. | Complicated: Implementation analysis, defining implementation strategies, implementation / project planning. All activities coordinated by project leaders of the Citrien program. | Complicated: Implementation analysis, defining implementation strategies, implementation / project planning. All activities coordinated by project leaders of the Citrien program. | Complicated: Implementation analysis, defining implementation strategies, implementation / project planning. All activities coordinated by project leaders of the Citrien program. | Complicated: Implementation analysis, defining implementation strategies, implementation / project planning. All activities coordinated by project leaders of the Citrien program. | Complicated: Implementation analysis, defining implementation strategies, implementation / project planning. All activities coordinated by project leaders of the Citrien program. |
| Domain 6 The wider system | | | | | | | | |
| 6A. What is the political, economic, regulatory, professional (e.g. medicolegal) and socio-cultural context for program roll-out? | Complex: Financial and regulatory requirements  raise professional, economic and legal  challenges | Complex: Financial and regulatory requirements  raise professional, economic and legal  challenges | Complex: Financial and regulatory requirements  raise professional, economic and legal  challenges | Complex: Financial and regulatory requirements  raise professional, economic and legal  challenges | Complex: Financial and regulatory requirements  raise professional, economic and legal  challenges | Complex: Financial and regulatory requirements  raise professional, economic and legal  challenges | Complex: Financial and regulatory requirements  raise professional, economic and legal  challenges | Complex: Financial and regulatory requirements  raise professional, economic and legal  challenges |
| Domain 7 Interaction and adaptation over time | | | | | | | | |
| 7A. How much scope is there for adapting and co-evolving the technology and the service over time? | Complex: Although there is a strong scope from the Citrien program, there are significant barriers to further  adaptation and/or coevolution of  the technology. | Complex: Although there is a strong scope from the Citrien program, there are significant barriers to further  adaptation and/or coevolution of  the technology. | Complex: Although there is a strong scope from the Citrien program, there are significant barriers to further  adaptation and/or coevolution of  the technology. | Complex: Although there is a strong scope from the Citrien program, there are significant barriers to further  adaptation and/or coevolution of  the technology. | Complex: Although there is a strong scope from the Citrien program, there are significant barriers to further  adaptation and/or coevolution of  the technology. | Complex: Although there is a strong scope from the Citrien program, there are significant barriers to further  adaptation and/or coevolution of  the technology. | Complex: Although there is a strong scope from the Citrien program, there are significant barriers to further  adaptation and/or coevolution of  the technology. | Complex: Although there is a strong scope from the Citrien program, there are significant barriers to further  adaptation and/or coevolution of  the technology. |
| 7B. How resilient is the organisation to handling critical events and adapting to unforeseen eventualities? | Simple: collective reflection,  and adaptive action are ongoing  and encouraged | Simple: collective reflection,  and adaptive action are ongoing  and encouraged | Simple: collective reflection,  and adaptive action are ongoing  and encouraged | Simple: collective reflection,  and adaptive action are ongoing  and encouraged | Simple: collective reflection,  and adaptive action are ongoing  and encouraged | Simple: collective reflection,  and adaptive action are ongoing  and encouraged | Simple: collective reflection,  and adaptive action are ongoing  and encouraged | Simple: collective reflection,  and adaptive action are ongoing  and encouraged |
