# Supplementary material for: The National Coordinated Citrien eHealth Program to Scale Up Telemonitoring: Protocol for a Before-and-After Evaluation Study
Source: JMIR Res Protoc. 2023 Jul 26;12:e45201. doi: 10.2196/45201 (PMC10413225; doi:10.2196/45201)
Supplement: Multimedia Appendix 2 [file resprot_v12i1e45201_app2.docx]

**Appendix 2. NASSS framework analysis of telemonitoring in antenatal care. (by example of Greenhalgh et al 2017, table 2)**

|  | Case A | Case B | Case C | Case D | Case E | Case F | Case G | Case H |
| --- | --- | --- | --- | --- | --- | --- | --- | --- |
| Domain 1. The condition or illness | | | | | | | | |
| 1A. Wat is the nature of the condition or illness? | Simple: Telemonitoring of blood pressure in pregnant women with elevated risk on pre-eclampsia at  home | Simple: Telemonitoring of blood pressure in pregnant women with elevated risk on pre-eclampsia at  home | Simple: Telemonitoring of blood pressure in pregnant women with elevated risk on pre-eclampsia at  home | Simple: Telemonitoring of blood pressure in pregnant women with elevated risk on pre-eclampsia at  home | Simple: Telemonitoring of blood pressure in pregnant women with elevated risk on pre-eclampsia at  home | Simple: Telemonitoring of blood pressure in pregnant women with elevated risk on pre-eclampsia at  home | Simple: Telemonitoring of blood pressure in pregnant women with elevated risk on pre-eclampsia at  home | Simple: Telemonitoring of blood pressure in pregnant women with elevated risk on pre-eclampsia at  home |
| 1B. What are the relevant socio-cultural factors and comorbidities? | Simple: Relatively young, good eHealth literacy | Simple: Relatively young, good eHealth literacy | Simple: Relatively young, good eHealth literacy | Simple: Relatively young, good eHealth literacy | Simple: Relatively young, good eHealth literacy | Simple: Relatively young, good eHealth literacy | Simple: Relatively young, good eHealth literacy | Simple: Relatively young, good eHealth literacy |
| Domain 2 The Technology | | | | | | | | |
| 2A. What are the key features of the technology? | Simple: A bloodpressure device with bluetooth linked to a smartphone, off the shelf, well tested, already in operation. | Simple: A bloodpressure device with bluetooth linked to a smartphone, off the shelf, well tested, already in operation. | Simple: A bloodpressure device with bluetooth linked to a smartphone, off the shelf, well tested, already in operation.. | Simple: Manually administred bloodpressure in patient portal. Not yet fully tested. | Complicated: A bloodpressure device with bluetooth linked to a smartphone, tested but not ready in operation. | Complicated: A bloodpressure device with bluetooth linked to a smartphone, not tested, not in operation. | Simple: A bloodpressure device with bluetooth linked to a smartphone, off the shelf, well tested, already in operation. | Simple: Manually administred bloodpressure in patient portal. Not yet fully tested. |
| 2B. What kind of knowledge does the technology bring into play? | Simple: Directly and transparently measures  changes in blood pressure | Simple: Directly and transparently measures  changes in blood pressure | Simple: Directly and transparently measures  changes in blood pressure | Simple: Directly and transparently measures  changes in blood pressure | Simple: Directly and transparently measures  changes in blood pressure selfmanage their lifestyle. | Simple: Directly and transparently measures  changes in blood pressure | Simple: Directly and transparently measures  changes in blood pressure | Simple: Directly and transparently measures  changes in blood pressure |
| 2C. What knowledge and/or support is required to use the technology? | Complicated: Medical and technical support by a medical service centre. Weekdays 9-5 support | Complicated: Different care planning and control in the outpatient clinic. Weekdays 9-5 support | Complicated: Different care planning and control in the outpatient clinic. Weekdays 9-5 support | Complicated: Different care planning and control in the outpatient clinic. Weekdays 9-5 support | Complicated: Different care planning and control in the outpatient clinic. Weekdays 9-5 support. | Complicated: Medical and technical support by a medical service centre. Weekdays 9-5 support. | Complicated: Medical and technical support by a medical service centre. Weekdays 9-5 support. | Complicated: Different care planning and control in the outpatient clinic. Weekdays 9-5 support |
| 2D. What is the technology supply model? | Simple: Third party and front-end integration in EHR. | Complicated: Third party and back-end integration in EHR. | Simple: Third party and front-end integration in EHR. | Complicated: Third party and back-end integration in EHR. | Complicated: Third party and front-end integration in EHR. | Simple: Third party and front-end integration in EHR. | Simple: Third party and front-end integration in EHR. | Complicated: Third party and back-end integration in EHR. |
| Domain 3 The value proposition | | | | | | | | |
| 3A. What is the developer’s business case for the technology (supply-side value)? | unknown | unknown | unknown | unknown | unknown | unknown | unknown | unknown |
| 3B. What is its desirability, efficacy, safety and cost-effectiveness (demand-side value)? | Simple: fewer emergency admissions, fewer hospital admissions, fewer regular check-up appointments, more self-management for the patient | Simple: fewer emergency admissions, fewer hospital admissions, fewer regular check-up appointments, more self-management for the patient | Simple: fewer emergency admissions, fewer hospital admissions, fewer regular check-up appointments, more self-management for the patient | Simple: fewer emergency admissions, fewer hospital admissions, fewer regular check-up appointments, more self-management for the patient | Simple: fewer emergency admissions, fewer hospital admissions, fewer regular check-up appointments, more self-management for the patient | Simple: fewer emergency admissions, fewer hospital admissions, fewer regular check-up appointments, more self-management for the patient | Simple: fewer emergency admissions, fewer hospital admissions, fewer regular check-up appointments, more self-management for the patient | Simple: fewer emergency admissions, fewer hospital admissions, fewer regular check-up appointments, more self-management for the patient |
| Domain 4 The adopter system (Staff, patient, lay carers) | | | | | | | | |
| 4A. What changes in staff roles, practices and identities are implied? | Complicated: less face-to-face contact, patient self-administered measurement, monitoring centralised, shift in tasks and skills of nurses | Complicated: less face-to-face contact, patient self-administered measurement, monitoring centralised, shift in tasks and skills of nurses | Complicated: less face-to-face contact, patient self-administered measurement, monitoring centralised, shift in tasks and skills of nurses | Complicated: less face-to-face contact, patient self-administered measurement, monitoring centralised, shift in tasks and skills of nurses nurses | Complicated: less face-to-face contact, patient self-administered measurement, monitoring centralised, shift in tasks and skills of nurses | Complicated: less face-to-face contact, patient self-administered measurement, monitoring centralised, shift in tasks and skills of nurses | Complicated: less face-to-face contact, patient self-administered measurement, monitoring centralised, shift in tasks and skills of nurses nurses | Complicated: less face-to-face contact, patient self-administered measurement, monitoring centralised, shift in tasks and skills of nurses |
| 4B. What input is expected of the patient (and/or immediate carer) – and is this achievable by, and acceptable to, them? | Complicated: Technical skills for device pairing, patient self-administered measurement, responsibility or self-management among patients = achievable and acceptable | Complicated: Technical skills for device pairing, patient self-administered measurement, responsibility or self-management among patients = achievable and acceptable | Complicated: Technical skills for device pairing, patient self-administered measurement, responsibility or self-management among patients = achievable and acceptable | Complicated: Technical skills for device pairing, patient self-administered measurement, responsibility or self-management among patients = achievable and acceptable | Complicated: Technical skills for device pairing, patient self-administered measurement, responsibility or self-management among patients = achievable and acceptable | Complicated: Technical skills for device pairing, patient self-administered measurement, responsibility or self-management among patients = achievable and acceptable | Complicated: Technical skills for device pairing, patient self-administered measurement, responsibility or self-management among patients = achievable and acceptable | Complicated: Technical skills for device pairing, patient self-administered measurement, responsibility or self-management among patients = achievable and acceptable |
| 4C. What is assumed about the extended network of lay carers? | Simple: Nothing in particular | Simple: Nothing in particular | Simple: Nothing in particular | Simple: Nothing in particular | Simple: Nothing in particular | Simple: Nothing in particular | Simple: Nothing in particular | Simple: Nothing in particular |
| Domain 5 The organization | | | | | | | | |
| 5A. What is the organization’s capacity to innovate? | Simple: Positive attitude management and healthcare providers. Sufficient (research) funding available for technology introduction Local champions in place. | Simple: Positive attitude management and healthcare providers. Sufficient (research) funding available for technology introduction Local champions in place. | Simple: Positive attitude management and healthcare providers. Sufficient (research) funding available for technology introduction Local champions in place. | Complicated: Positive attitude management and healthcare providers. Local champions in place. But limited resources. | Simple: Positive attitude healthcare providers. Sufficient (research) funding available for technology introduction Local champions in place. | Simple: Positive attitude healthcare providers. Sufficient (research) funding available for technology introduction Local champions in place. | Simple: Positive attitude management and healthcare providers. Sufficient (research) funding available for technology introduction Local champions in place. | Complicated: Positive attitude management and healthcare providers. Local champions in place. But limited resources. |
| 5B. How ready is the organization for this technology-supported change? | Simple: Organisation-wide innovation programme available at inception. A readiness analysis has been conducted but not openly available. | Simple: Organisation-wide innovation programme available at inception. | Simple: Organisation-wide innovation programme available at inception. A readiness analysis has been conducted but not openly available. | Complicated: Organisation-wide innovation programme not available at inception. A readiness analysis has not been conducted. | Simple: Organisation-wide innovation programme available at inception. A readiness analysis has been conducted but not openly available. | Complicated: Organisation-wide innovation programme not available at inception. A readiness analysis has not been conducted. | Simple: Organisation-wide innovation programme available at inception. A readiness analysis has been conducted but not openly available. | Complicated: Organisation-wide innovation programme not available at inception. A readiness analysis has not been conducted. |
| 5C. How easy will the adoption and funding decision be? | Complicated: no structural reimbursement, cost-benefit balance neutral or unknown. | Complicated: no structural reimbursement, cost-benefit balance neutral or unknown. | Complicated: no structural reimbursement, cost-benefit balance neutral or unknown. | Complicated: no structural reimbursement, cost-benefit balance neutral or unknown. | Complicated: no structural reimbursement, cost-benefit balance neutral or unknown. | Complicated: no structural reimbursement, cost-benefit balance neutral or unknown. | Complicated: no structural reimbursement, cost-benefit balance neutral or unknown. | Complicated: no structural reimbursement, cost-benefit balance neutral or unknown. |
| 5D. What changes will be needed in team interactions and routines? | Complicated: New routines and care pathways, new communication and escalation protocols, but readily aligned. | Complicated: New routines and care pathways, new communication and escalation protocols, but readily aligned. | Complicated: New routines and care pathways, new communication and escalation protocols, but readily aligned. | Complicated: New routines and care pathways, new communication and escalation protocols, but readily aligned. | Complicated: New routines and care pathways, new communication and escalation protocols, but readily aligned. | Complicated: New routines and care pathways, new communication and escalation protocols, but readily aligned. | Complicated: New routines and care pathways, new communication and escalation protocols, but readily aligned.. | Complicated: New routines and care pathways, new communication and escalation protocols, but readily aligned. |
| 5E. What work is involved in implementation and who will do it? | Complicated: Implementation analysis, defining implementation strategies, implementation / project planning. All activities coordinated by project leaders of the Citrien program. | Complicated: Implementation analysis, defining implementation strategies, implementation / project planning. All activities coordinated by project leaders of the Citrien program. | Complicated: Implementation analysis, defining implementation strategies, implementation / project planning. All activities coordinated by project leaders of the Citrien program. | Complicated: Implementation analysis, defining implementation strategies, implementation / project planning. All activities coordinated by project leaders of the Citrien program. | Complicated: Implementation analysis, defining implementation strategies, implementation / project planning. All activities coordinated by project leaders of the Citrien program. | Complicated: Implementation analysis, defining implementation strategies, implementation / project planning. All activities coordinated by project leaders of the Citrien program. | Complicated: Implementation analysis, defining implementation strategies, implementation / project planning. All activities coordinated by project leaders of the Citrien program. | Complicated: Implementation analysis, defining implementation strategies, implementation / project planning. All activities coordinated by project leaders of the Citrien program. |
| Domain 6 The wider system | | | | | | | | |
| 6A. What is the political, economic, regulatory, professional (e.g. medicolegal) and socio-cultural context for program roll-out? | Complex: Financial and regulatory requirements  raise professional, economic and legal  challenges | Complex: Financial and regulatory requirements  raise professional, economic and legal  challenges | Complex: Financial and regulatory requirements  raise professional, economic and legal  challenges | Complex: Financial and regulatory requirements  raise professional, economic and legal  challenges | Complex: Financial and regulatory requirements  raise professional, economic and legal  challenges | Complex: Financial and regulatory requirements  raise professional, economic and legal  challenges | Complex: Financial and regulatory requirements  raise professional, economic and legal  challenges | Complex: Financial and regulatory requirements  raise professional, economic and legal  challenges |
| Domain 7 Interaction and adaptation over time | | | | | | | | |
| 7A. How much scope is there for adapting and co-evolving the technology and the service over time? | Simple: there is a strong scope from the Citrien program to embed the technology. | Simple: there is a strong scope from the Citrien program to embed the technology. | Simple: there is a strong scope from the Citrien program to embed the technology. | Complicated: Although there is a strong scope from the Citrien program to embed the technology, there is potential for coevolving the technology | Simple: there is a strong scope from the Citrien program to embed the technology. | Simple: there is a strong scope from the Citrien program to embed the technology. | Simple: there is a strong scope from the Citrien program to embed the technology. | Complicated: Although there is a strong scope from the Citrien program to embed the technology, there is potential for coevolving the technology |
| 7B. How resilient is the organisation to handling critical events and adapting to unforeseen eventualities? | Simple: collective reflection,  and adaptive action are ongoing  and encouraged | Simple: collective reflection,  and adaptive action are ongoing  and encouraged | Simple: collective reflection,  and adaptive action are ongoing  and encouraged | Simple: collective reflection,  and adaptive action are ongoing  and encouraged | Simple: collective reflection,  and adaptive action are ongoing  and encouraged | Simple: collective reflection,  and adaptive action are ongoing  and encouraged | Simple: collective reflection,  and adaptive action are ongoing  and encouraged | Simple: collective reflection,  and adaptive action are ongoing  and encouraged |
